# Lactic Acid Bacteria from Gamecock and Goat Originating from Phitsanulok, Thailand: Isolation, Identification, Technological Properties and Probiotic Potential

**DOI:** 10.4014/jmb.2110.10040

**Published:** 2022-01-13

**Authors:** Noraphat Hwanhlem, Lakha Salaipeth, Rangsun Charoensook, Pochanart Kanjan, Suppasil Maneerat

**Affiliations:** 1 Division of Animal Science and Feed Technology, Department of Agricultural Science, Faculty of Agriculture, Natural Resources and Environment, Naresuan University, Phitsanulok 65000, Thailand; 2 School of Bioresources and Technology, King Mongkut’s University of Technology Thonburi, Bangkok 10150, Thailand; 3 Department of Agricultural and Fishery Science, Faculty of Science and Technology, Prince of Songkla University, Pattani, 94000 Thailand; 4 Center of Excellence in Innovative Biotechnology for Sustainable Utilization of Bioresources, Faculty of Agro-Industry, Prince of Songkla University, Hat Yai, Songkhla 90110, Thailand

**Keywords:** Lactic acid bacteria, gamecock, goat, pathogenic bacteria, probiotic

## Abstract

From independent swab samples of the cloaca of indigenous gamecocks (CIG), anus of healthy baby goats (AHG), and vagina of goats (VG) originating from Phitsanulok, Thailand, a total of 263 isolates of lactic acid bacteria (LAB) were collected. Only three isolates, designated C707, G502, and V202, isolated from CIG, AHG, and VG, respectively, exhibited an excellent inhibitory zone diameter against foodborne pathogenic bacteria when evaluated by agar spot test. Isolates C707 and G502 were identified as *Enterococcus faecium*, whereas V202 was identified as *Pediococcus acidilactici*, based on 16S rRNA sequence analysis. When foodborne pathogenic bacteria were co-cultured with chosen LAB in mixed BHI-MRS broth at 39&deg;C, their growth was suppressed. These LAB were found to be capable of surviving in simulated stomach conditions. Only the isolate G502 was able to survive in the conditions of simulated intestinal juice. This research suggests that selected LAB could be used as a food/feed supplement to reduce foodborne pathogenic bacteria and improve the safety of animal-based food or feed.

## Introduction

Food safety is becoming increasingly important to both producers and consumers. Food safety, according to Olson and Slack [1], begins on the farm. The system for producing animals, on the other hand, is related to the larger issue of antibiotic usage in food animals having negative health impacts on consumers [2]. As a result, many producers struggle to offer them with food safety assurance through regulation. They are also becoming more aware of their responsibilities in this area, as well as the importance of collaborating with other sectors of the agri-food and livestock industries. However, antibiotics are still used in intensive livestock farming to improve feed efficiency, animal growth performance, and the quality of animal products, which stimulates the spread of resistant bacteria and the development of antibiotic resistance. Antibiotics are given in a variety of ways, including medicated feed additives, depot injections, and drinking water [3]. Humans can acquire resistant bacteria from livestock production through contaminated eggs, meat, and dairy products, direct contact with animals, and the environment. Antibiotics in farmed animals may pose a risk not only to the health of consumers but also to the environment. This is a critical issue in the oncoming public health crisis, because natural genetic variation in bacterial populations and individual organisms can spread mutations that render antibiotics ineffective while providing a protective effect to the mutated bacterium.

Biocontrol is a technology that uses the activity of microorganisms to inhibit the growth of microbial spoilage and pathogens to extend shelf life and promote food safety [4, 5]. This biological technique aims to reduce the use of chemical additions such as nitrite, sodium chloride, organic acids, and antibiotics in food and feed. The antagonistic activities of lactic acid bacteria (LAB) against bacterial spoilage and pathogens have been the focus of most bio-preservation research [6]. The European Food Safety Authority (EFSA) has given certain LAB strains the “Qualified Presumption of Safety” (QPS) classification. According to Brashears *et al*. [7], the use of LAB in animal feeding could improve food safety since their protective actions can prevent foodborne pathogenic bacteria in a live animal before slaughter. Due to customer demand for products with minimal antibiotics and chemical preservatives, the use of microorganisms to improve food and feed safety has gained interest in recent years. LAB not only produce antimicrobial compounds like lactic acid, fatty acids, hydrogen peroxide (H_2_O_2_), diacetyl, bacteriocins, and antifungal peptides to control bacterial pathogens or spoilage, but they also compete with other species by acidifying their environment and rapidly depleting available nutrients [8]. However, not all LAB will reduce foodborne pathogens in farm animals, but carefully selected strains administered under the right conditions can help reduce food spoilage and pathogenic bacteria in farm animals and the livestock production industry.

Because of the host species specificity, probiotics targeted to food or feed additives are typically derived from food animals [9]. This allows the probiotics to better adapt to the circumstances of the animal gastrointestinal tract (GIT) and the animal-based food product. Therefore, the objectives of this study were to isolate, select, identify, and evaluate the technological properties and probiotic potential of LAB from the cloaca of male indigenous gamecocks, vagina of goats, and anus of one-month (mixed-sex) healthy baby goats originating from Phitsanulok, one of the most important agricultural areas in lower northern Thailand that has yet to be studied. This study shows that appropriate LAB has some probiotic potential for application as a food/feed additive in the food/feed industry.

## Materials and Methods

### Sample Collection and Isolation of LAB

Swab samples were collected from the cloaca of 10 male indigenous gamecocks, the vagina of 10 goats and the anus of 10 one-month (mixed-sex) healthy baby goats originating in Phitsanulok, and kept in 5 ml of sterile physiological saline solution (SPSS) (0.85% NaCl, 0.1% peptone) and transported to the laboratory. Appropriate decimal dilutions (10^-1^-10^-5^) were then prepared in SPSS, poured into sterile Petri dishes on de Man, Rogosa and Sharpe agar (MRS) (Himedia, India) containing 0.004% bromocresol purple (w/v) (Ajax finechem, Australia) and left to dry for 15 min at room temperature under sterile condition. Plates were overlaid with 10 ml of 1%bacteriological agar (Himedia, India) and subjected to the incubator for 24-48 h at 39°C (according to the body temperature of the tested animal). The bacterial colonies which exhibited the color of the agar from purple to yellow were individually picked and streaked on MRS agar containing 0.004% bromocresol purple (w/v). To purify the isolates, this process was repeated. Each of the isolates was first tested for catalase by placing a drop of 3% H_2_O_2_ solution on the purified isolate colony. Immediate formation of bubbles indicated the presence of catalase in the cells. Only catalase-negative isolates were Gram-staining tested, and only Gram-positive isolates were chosen and kept at −20°C in MRS broth with 30% glycerol. Strains were sub-cultured twice in MRS broth for 24-48 h at 39°C for routine analysis [9].

### Determination of Sensitivity of Foodborne Pathogenic Bacteria and Related LAB to Isolated LAB

The sensitivity of foodborne pathogenic bacteria and related LAB to isolated LAB was determined by the agar spot test.

**Indicator bacterial strains.** The foodborne pathogenic bacteria (*Escherichia coli* CIP 76.24, *Listeria innocua* CIP 80.11^T^, *Salmonella* Typhimurium CIP 58.58, and *Staphylococcus aureus* CIP 76.25) were obtained from Oniris (Ecole Nationale Nantes Atlantique Vétérinaire, Agroalimentaire et de l’Alimentation, France). Related LAB (*Lactobacillus sakei* subsp. *sakei* JCM 1157) was obtained from Japan Collection of Microorganisms. Growth media and growth condition of bacterial indicators are summarized in Table 1.

**Agar spot test.** The sensitivity of foodborne pathogenic bacteria and related LAB was determined by agar spot test. One microliter of overnight culture (6 log CFU/ml) of isolated LAB and LAB used as positive control [10] was dropped onto the surface of MRS agar plates (1.5% agar) and left to dry under sterile conditions at room temperature for 15 min. The plates were then overlaid with 10 ml of Brain heart infusion (BHI) (Himedia, India) or MRS soft agar (1% agar) containing foodborne pathogenic bacteria or related LAB (6 log CFU/ml), respectively. Plates were proceeded to the incubator at the optimum temperature of each indicator strain for 24 h and subsequently checked for inhibition zone diameters.

### Identification and Phylogenetic Analysis of the Selected LAB

16S rRNA sequence analysis of selected LAB was carried out by Macrogen's sequencing service, Macrogen, Inc.(Seoul, Korea). The obtained sequences were compared with those available in GenBank database, using the Basic Local Alignment Search Tool (BLAST) at the National Center of Biotechnology Information website (http://www.ncbi.nlm.nih.gov). Sequences were analyzed and edited using the program DNAMAN. All sequences were grouped into operational taxonomic units (OTUs) at a threshold of 98% sequence identity and then aligned by using MUSCLE within the MEGA package, version 7.0 [11]. The evolutionary history was inferred using the Neighbor-Joining method [12]. The percentage of replicate trees in which the associated taxa clustered together in the bootstrap test (1000 replicates) are shown next to the branches [13]. The tree is drawn to scale, with branch lengths in the same units as those of the evolutionary distances used to infer the phylogenetic tree. The evolutionary distances were computed using the Maximum Composite Likelihood method and are in the units of the number of base substitutions per site. Codon positions included were 1st+2nd+3rd+Noncoding. All positions containing gaps and missing data were eliminated. Evolutionary analyses were conducted in MEGA7.

### Co-Cultivation of Selected LAB and Foodborne Pathogenic Bacteria in Mixed BHI-MRS Broth at 39°C

The efficacy of selected LAB to inhibit the growth of *E. coli* CIP 76.24, *L. innocua* CIP 80.11^T^ and *S*. Typhimurium CIP 58.58 was performed by a co-cultivation in mixed BHI-MRS broth at 39°C (according to the body temperature of the isolation source (gamecock and goat) of LAB). Two-time concentration (2×conc.) of BHI and MRS was prepared according to the manufacturer's recommendation and then autoclaved. Mixed BHI-MRS broth was prepared by mixing sterile BHI (2×conc.) and sterile MRS (2×conc.) broth in a 1:1 ratio (v/v). The co-cultivation batches including three different culture treatments, treatment 1 corresponded to the co-inoculation of *E. coli* CIP 76.24 and G502 (EC+G502); treatment 2, the co-inoculation of *L. innocua* CIP 80.11^T^ and V202 (LI+V202); and treatment 3, the co-inoculation of *S*. Typhimurium CIP 58.58 and C707 (ST+C707). For each indicator strain tested by co-cultivation, an individual culture was also conducted as the control treatment (EC, LI, and ST), as well as for the selected LAB (C707, G502, and V202). For each co-inoculation treatment, sterile mixed BHI-MRS broth (49 ml) was delivered to a 50-ml test tube and then 500 μl of indicator sub-cultured in BHI broth (4-5 log CFU/ml) was inoculated to the tube. Instantly, 500 μl of the selected LAB sub-cultured in MRS broth (4-5 log CFU/ml) was inoculated aseptically to the same tube. For the control treatments, subcultures of indicator strains and selected LAB were individually inoculated into the tube containing 49.5 ml of sterile mixed BHI-MRS broth and then were incubated at 39°C for 24 h. Aliquots from each tube were analyzed at 0, 3, 6, 12, 18 and 24 h for the growth of selected LAB and indicator strains. A total count for individual LAB and the tested indicator strains was evaluated by preparing 10-fold serial dilutions in SPSS. An inoculum (0.1 ml) of each appropriate dilution was spread onto selective agar. *E. coli* CIP 76.24 was enumerated onto MacConkey Agar (Himedia, India), *L. innocua* CIP 80.11^T^ onto *Listeria* Selective Agar Base (Himedia, India) and *S*. Typhimurium CIP 58.58 onto SS Agar (Salmonella Shigella Agar) (Himedia, India). In addition, the growth of selected LAB was checked on MRS agar containing 0.004% bromocresol purple (w/v). Plates were incubated at the optimum temperature of each strain (Table 1), colonies were then enumerated, and the results were expressed as log CFU/ml [14].

### Survival of Selected LAB in Simulated Gastrointestinal Tract (GIT) Condition

**Survival of selected LAB in simulated stomach condition.** The selected LAB (C707, G502, and V202) were cultivated in MRS broth at 39°C for 24 h. The cultures were centrifuged at 6,904 g for 10 min at 4°C to separate the supernatant and bacterial cell. Bacterial cell was washed twice with SPSS before being resuspended in 50 mM potassium phosphate buffer saline (PBS, 50 mM K_2_HPO_4_, 50 mM KH_2_PO_4_, pH 7.2), adjusted to pH 3 with 1 M of NaOH and HCl, added with 3 mg/ml pepsin (Fluka, Steinheim), and incubated at 39°C for 3 h. Tolerant LAB were assessed in terms of viable colony counts, enumerated by drop plate method on MRS agar, incubated at 39°C for 24 h, and expressed as log CFU/ml [10].

**Survival of selected LAB in simulated intestinal juice.** After 3 h being subjected to simulated stomach condition, LAB were centrifuged at 6,904 g for 10 min at 4°C and washed twice with SPSS before being resuspended in PBS solution, pH 8 containing 1 mg/ml pancreatin (Sigma-Aldrich, USA), 1 mg/ml trypsin (Sigma-Aldrich, USA), 1 mg/ml α-chymotrypsin (Sigma-Aldrich, USA), and 5 mg/ml bile salts (Oxoid, England), and incubated at 39°C for 3 h. Tolerant LAB were assessed in terms of viable colony counts, enumerated by drop plate method on MRS agar, incubated at 39°C for 24 h and expressed as log CFU/ml [10].

### Statistical Analysis

All data were analyzed by one-way analysis of variance (one-way ANOVA) using the Statistical Package for the Social Sciences (SPSS), version 17.0 (SPSS Inc., USA). All results were presented as mean ± S.D. Differences among the treatments were performed using Duncan's multiple range test. Probabilities (*p* < 0.05) were considered significant.

## Results and Discussion

### Isolation of LAB from Cloaca of Indigenous Gamecocks, Vagina of Goats and Anus of Healthy Baby Goats

For LAB isolation, swabs from the cloaca of indigenous gamecocks (CIG), the anus of healthy baby goats (AHG), and the vagina of goats (VG) from Phitsanulok, Thailand were used. On MRS agar containing 0.004%bromocresol purple, 304 isolates producing acid bacteria (110, 102, and 92 isolates from CIG, AHG, and VG, respectively) were isolated (data not shown). Bromocresol purple, which has a yellow color below pH 5.3 and a purple color above pH 6.7, was utilized as a pH change indicator for the detection of acid-producing bacterial strains [15, 16]. Using the Gram-positive and catalase-negative criteria, 263 of the 304 isolates were confirmed as LAB.

### Determination of Sensitivity of Foodborne Pathogenic Bacteria and Related LAB to Isolated LAB

Agar spot test was used to determine the sensitivity of foodborne pathogenic bacteria and related LAB to isolated LAB and LAB used as positive control. Among 263 isolates of isolated LAB, only 13 isolates had an excellent inhibitory zone diameter against foodborne pathogenic bacteria including *E. coli* CIP 76.24, *L. innocua* CIP 80.11^T^, *S*. Typhimurium CIP 58.58, and *S. aureus* CIP 76.25. Nine isolates of them could also suppress related LAB (*L. sakei* subsp. *sakei* JCM 1157) as shown in Table 2. Foodborne pathogenic bacteria contamination in food and feed is a critical problem that causes a wide range of diseases with significant public health and economic consequences worldwide [17]. Food animals and animal-based food products are the key reservoirs and vehicles of transmission for many food-borne zoonotic bacterial diseases, according to Abebe *et al*. [18]. *Campylobacter* sp., *E. coli*, *L. monocytogenes*, *Salmonella* sp., and *S. aureus* are the most common zoonotic bacterial pathogens which cause a foodborne illness associated with consumption of unhygienic or contaminated animal products. Healthcare-associated infections can be caused by *E. coli*. It is the most common cause of urinary tract infections and is usually blamed for diarrhea in people who travel internationally. Infection with *E. coli* O157:H7 is a major public health hazard. Despite the fact that the total number of *E. coli* O157:H7 infections is lower than other enteric pathogens like *Salmonella* or *Campylobacter* spp., the diseases caused by *E. coli* O157:H7 have substantially higher hospitalization and death rates. Human infection induced by *E. coli* O157:H7 can have a wide range of symptoms, from asymptomatic to fatal. The majority of cases begin with severe stomach cramps, non-bloody diarrhea, and vomiting, and self-resolve without further complications. Some persons may develop a fever, which is usually mild (less than 38.5ºC). The majority of people recover in 5-7 days. In 1-3 days, however, some individuals develop bloody diarrhea or hemorrhagic colitis (HC). The condition can proceed to life-threatening consequences, such as hemolytic uremic syndrome or thrombocytopenic purpura, in 5-10% of HC patients [19-21]. *Listeria* infections are most common in newborns, the elderly, pregnant women, and immunocompromised people. People who do not have these risk factors, however, can be affected. Infection with *L. monocytogenes* causes a self-limiting gastrointestinal infection with fever and diarrhea in healthy people. In healthy people, *L. monocytogenes* infection normally results in self-limited febrile diarrhea or is asymptomatic; however, in immunocompromised people, it can result in clinical episodes of invasive listeriosis. Listeriosis can strike at any time or in outbreaks. Infection can induce a wide range of symptoms, from feverish gastroenteritis to invasive diseases such bacteremia, sepsis, and meningoencephalitis [22, 23]. Infection with *Salmonella* spp. is one of the primary causes of morbidity, acute diarrheal disease, and mortality around the world, with the host immune response varied depending on whether the infection is acute and localized or systemic and persistent. Salmonellosis can range in severity from simple gastroenteritis (diarrhea, abdominal cramps, and fever) to enteric fevers (including typhoid fever), which are life-threatening febrile systemic infections that require rapid antibiotic treatment. There are sporadic infections and an asymptomatic carrier condition. Self-limited, simple gastroenteritis is the most prevalent form of salmonellosis [24-26]. *S. aureus* is a commensal bacterium as well as an important opportunistic human pathogen, causing bacteremia, infective endocarditis, skin and soft tissue infections (impetigo, folliculitis, furuncles, carbuncles, cellulitis, scalded skin syndrome, and others), osteomyelitis, septic arthritis, prosthetic device infections, pulmonary infections (pneumonia and empyema). These bacteria can induce invasive infections and/or toxin-mediated illnesses, depending on the strains involved and the site of infection [27-29].

LAB derived from animal origin has been shown to have antimicrobial properties against a variety of foodborne pathogenic bacteria, including LAB isolated from poultry feces [30], pig feces [31], and sheep milk [32]. LAB play a significant role in rapidly-produce copious amounts of organic acid end products, resulting in a pH drop in the fermentation. Diacetyl and H_2_O_2_ can also contribute to control undesirable microorganisms. LAB also produces bacteriocins, which are antimicrobial peptides synthesized on ribosomes during the early growth phase, to protect inhabitants, reduce the number of competitors, and obtain more nutrients and living space in environments [33, 34]. The amounts and types of different compounds produced during fermentation, on the other hand, are dependent on the LAB strain, growth conditions, and culture compositions. Organic acids created cause a drop in pH and the generation of undissociated forms of molecules. Low external pH induces acidification of the cell cytoplasm, while the undissociated acid can diffuse passively across the membrane since it is lipophilic. The undissociated acid disrupts substrate transport mechanisms by lowering the electrochemical proton gradient or modifying cell membrane permeability. H_2_O_2_ is another antimicrobial molecule produced by LAB in the presence of oxygen, with effects such as denaturing of certain enzymes due to oxidation of sulfhydryl groups and increased membrane permeability due to peroxidation of membrane lipids [10, 14, 35].

Thirteen isolates of LAB exhibiting an excellent inhibition zone diameter against foodborne pathogenic bacteria were obtained. However, for further studies, we have decided to select only one strain from each isolation source based on the criteria of the highest inhibition zone against the pathogenic bacterial indicator. The results suggest that LAB C707, G502, and V202, all of which were isolated from animals—CIG, AHG, and VG, respectively-had better activity against foodborne pathogenic bacteria than other isolates. They were finally chosen for further study.

### Identification and Phylogenetic Analysis of the Selected LAB

**Fig. 1:**
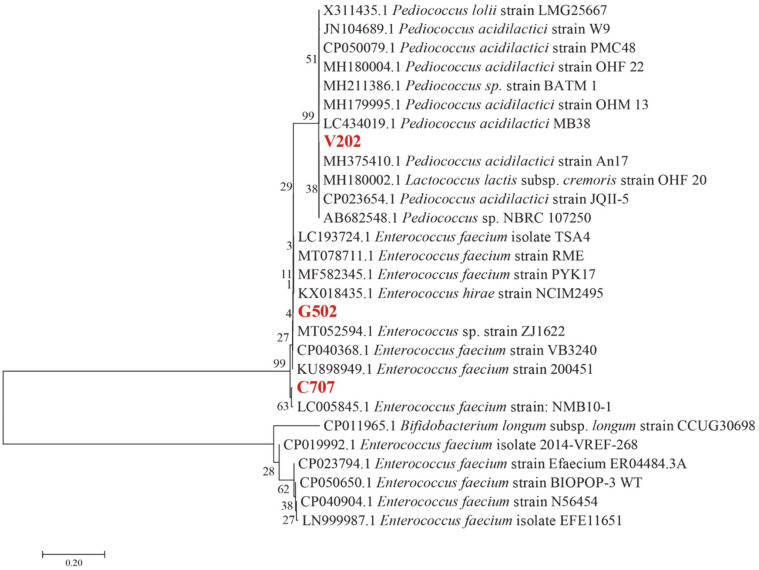
Neighbor-joining phylogenetic tree based on 16S rRNA gene sequences, showing the positions of strain C707, G502, and V202 and some related taxa deposited in Genbank. Bootstrap percentages (based on 1000 replications) are shown at branch points. The scale bar represents the expected percentage of substitutions per nucleotide position.

The identification results and neighbor-joining phylogenetic tree based on 16S rRNA gene sequence analysis of selected LAB are shown in Table 3 and Fig. 1, respectively. Isolates C707 and G502 were identified as *Enterococcus faecium*. Isolate V202 was identified as *Pediococcus acidilactici*. The obtained sequences were deposited in Genbank as accession numbers MW857160, MW857159, and MW857158, respectively. *Enterococcus* and *Pediococcus* are Gram-positive bacteria that belong to LAB, which are non-spore-forming cocci, catalase-negative, non-motile, and formed circular and off-white colonies on MRS agar [36].

Enterococci are widespread bacteria that have a prevalent habitat in the gastrointestinal tract of humans and animals. They may also be found in the environment or complex ecosystems such as food, feed, soil, plants, water, and agro-industrial waste [37]. These bacteria have been used as probiotics in the food and feed industries. Several bacteriocins (enterocins)-antimicrobial peptides-are produced simultaneously by some enterococci strains, giving them a competitive advantage over other bacteria for colonization and niche control. They are not in the list of the QPS due to some of them are known to be opportunistic pathogens. They are also frequently associated with nosocomial infections and have been implicated in human diseases such as bacteremia, endocarditis, and urinary tract infections [38]. Some of them however, have been proven to be used as a probiotic. Shi *et al*. [39] reported that *E. faecium* MK-SQ-1 isolated from chicken bile inhibited the growth of *Salmonella* Enteritidis and *S. aureus*. The genes responsible for virulence including *asa1*, *cylA*, *efaA*, *esp*, *gelE*, and *hyl* of this strain were not detected. The mice administered orally with a very high dose (2 × 10^9^ CFU) of the strain daily for 35 days were confirmed to be normal. By giving the strain to mice every day for 21 days, it dramatically increased their blood IgG level and phagocytic index (*p* < 0.05). Moreover, the strain did not have multi-antibiotic resistance and vancomycin resistance. These data suggest that some *Enterococcus* without the virulence factors and antibiotic resistance gene do not pose a threat to human or animal health. As a result, they could be used as probiotics or bioprotective cultures in the food and feed industries.

Pediococci are Gram-positive spherical cocci forming pairs and tetrads but not chains. They are catalase-negative, non-spore-forming, and non-motile facultative anaerobes. They are considered as a potential probiotic candidate due to their ability to survive, adhere to the GIT, and immune modulation capability [40, 41]. *P. acidilactici* strains have been found in several different samples, including cheese product [36], milk [42], meat product [43], and broiler chickens [44]. It is a possible LAB used as starter culture in dairy, meat, and vegetable fermentation which cause characteristic flavor changes, enhance hygienic quality and prolong the shelf life of various products. It contributes to food biopreservation by producing bacteriocins such as pediocins A [45], PA-1/AcH [46], and L50 [47] which are active against a wide range of Gram-positive bacteria (*Bacillus subtilis*, *L. monocytogenes*, *S. aureus*, *Clostridium perfringens*, *C. botulinum*, and *Mycobacterium smegmatis*) as well as Gram-negative bacteria (*E. coli*, and *Proteus vulgaris*) [36, 41, 48].

### Co-Cultivation of Selected LAB and Foodborne Pathogenic Bacteria in Mixed BHI-MRS Broth at 39°C

**Fig. 2:**
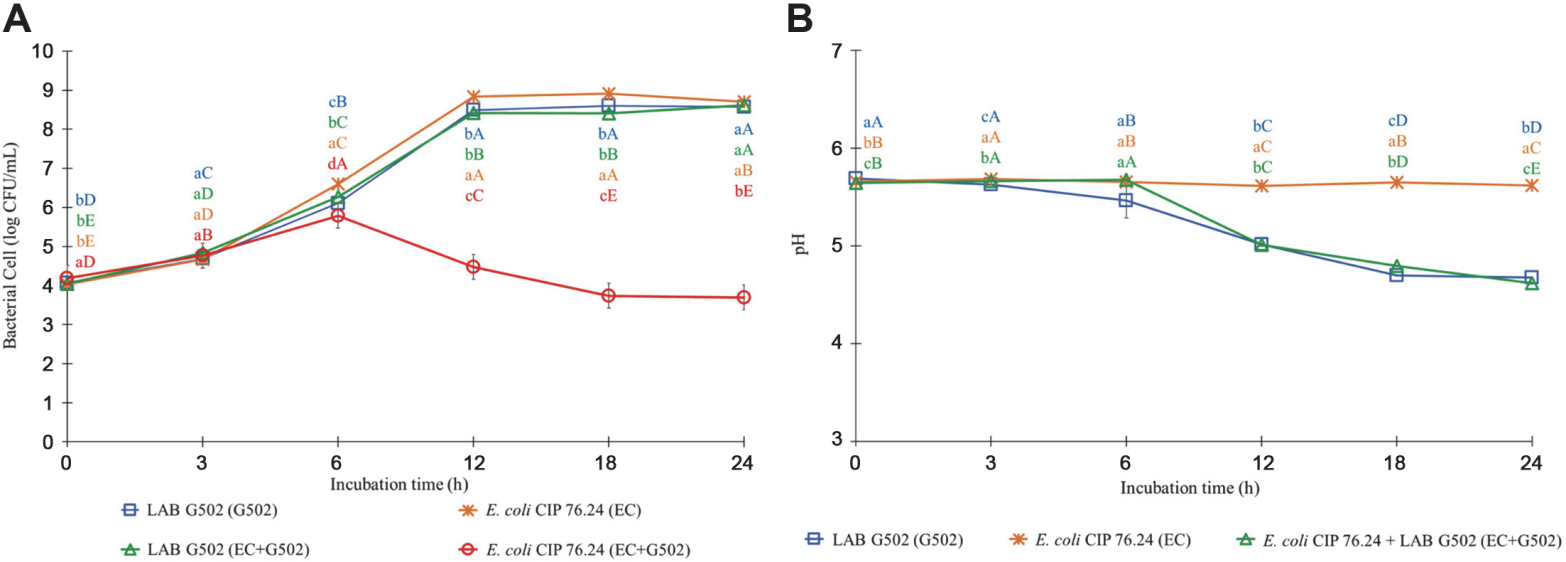
The growth curves of *Escherichia coli* CIP 76.24 and LAB G502 (2A), and the changes in pH of culture media (2B) when single cultivated and co-cultivated in mixed BHI-MRS broth at 39°C for 24 h. Error bars represent the standard deviation of the mean value. Each color of the alphabet represents each treatment. Different lowercase letters with different colors (a-d) at the same time point and different uppercase letters with the same color (**A-E**) indicate significant differences (*p* < 0.05) between treatments and within treatment, respectively.

The growth of *E. coli* CIP 76.24 co-cultivated with isolate G502 (EC+G502) at 39°C slightly increased during the first 6 h, then decreased slightly until 24 h of incubation, and finally remained at 3.69 ± 0.04 log CFU/ml (Fig. 2A). Consumers are concerned about the emergence and spread of Enterobacteriaceae, particularly *E. coli*, from animal origins or animal-based food products [49]. *E. coli* is a zoonotic pathogen that can cause serious health problems in humans and animals. It is one of a number of causes of diarrhea in sheep, goats, chickens, and cows that can be found all over the world. There is a high incidence associating with intensive rearing of kids under conditions of overcrowding and poor sanitation. It is also a serious problem of public health significance where the organism is responsible for foodborne illness [50-52].

**Fig. 3:**
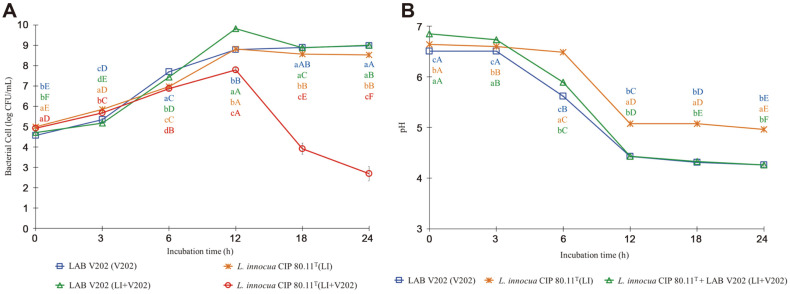
The growth curves of *Listeria innocua* CIP 80.11^T^ and LAB V202 (3A), and the changes in pH of culture media (3B) when single cultivated and co-cultivated in mixed BHI-MRS broth at 39°C for 24 h. Error bars represent the standard deviation of the mean value. Each color of the alphabet represents each treatment. Different lowercase letters with different colors (a-d) at the same time point and different uppercase letters with the same color (**A-F**) indicate significant differences (*p* < 0.05) between treatments and within treatment, respectively.

The growth of *L. innocua* CIP 80.11^T^ co-cultivated with the isolate V202 (LI+V202) increased during the first 12 h of incubation, then drastically decreased after 12 h, and remained at 2.69 ± 0.36 log CFU/ml at 24 h of incubation (Fig. 3A). *L. innocua* CIP 80.11^T^ was used as a nonpathogenic surrogate for *L. monocytogenes* [53], a Gram-positive, non-spore-forming, catalase-positive bacterium, recognized as a concerned foodborne pathogen. It causes listeriosis, a foodborne zoonosis that can be transmitted to humans via infected animals such as sheep, cattle, goats, and infrequently pigs, as well as through the consumption of certain animal-based foods [22, 54].

**Fig. 4:**
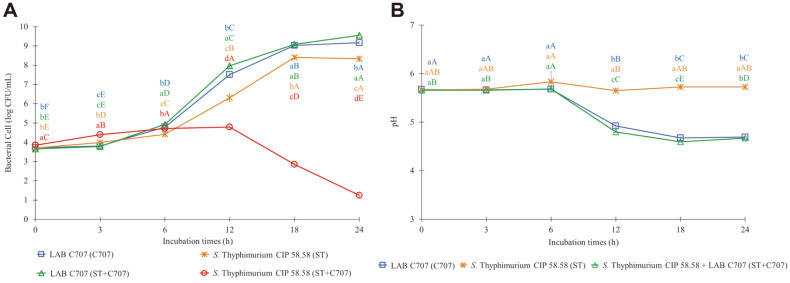
The growth curves of *Samonella* Typhimurium CIP 58.58 and LAB C707 (4A), and the changes in pH of culture media (4B) when single cultivated and co-cultivated in mixed BHI-MRS broth at 39°C for 24 h. Error bars represent the standard deviation of the mean value. Each color of the alphabet represents each treatment. Different lowercase letters with different colors (a-d) at the same time point and different uppercase letters with the same color (**A-F**) indicate significant differences (*p* < 0.05) between treatments and within treatment, respectively.

The growth of *S*. Typhimurium CIP 58.58 co-cultivated with the isolate C707 (ST+C707) remained stable during the first 12 h of incubation, then dramatically decreased after 12 h, and finally remained at 1.25 ± 0.12 log CFU/ml as shown in Fig. 4A. *Salmonella* spp.is a Gram negative intracellular enteric bacterium belonging to the family of Enterobacteriaceae. Different serovars were considered responsible for disease outbreaks of public health concern. However, *S*. Typhimurium and *S. Enteritidis* are the most common causes of foodborne illness worldwide. Salmonellosis, which is caused by *Salmonella* spp., is one of the most common zoonotic infections in poultry flocks. The majority of human diseases are linked to the consumption of eggs and chicken meat, which are typically contaminated during the slaughtering process and spread throughout the food chain. Salmonellosis in chicken also causes significant costs in the poultry industry in terms of mortality, poor growth, and egg production reduction [55-57].

The amounts of *E. coli* CIP 76.24 (EC), *L. innocua* CIP 80.11^T^(LI), *S*. Typhimurium CIP 58.58 (ST), and selected LAB (C707, G502 and V202) in the control treatments, and selected LAB in co-cultivations (EC+G502, LI+V202, ST+C707) increased during the first 3-12 h, and then the counts remained stable about 8-9 log CFU/ml throughout the incubation till 24 h.

The findings of our investigation suggest that selected LAB (C707, G502, and V202) can suppress foodborne pathogenic bacteria, which corresponds to pH drops as seen in Figs. 2B, 3B, and 4B. The decrease in pH during the first 12 h of incubation, on the other hand, had no effect on the proliferation of those foodborne pathogenic bacteria. After 6-12 h of incubation, the pH of all treatments cultured with selected LAB dropped considerably and remained steady at 4.3-4.6 until the end of the experiments.

Gao *et al*. [17] addressed that the main possible substances to the inhibitory effects of LAB consist of acid, H_2_O_2_, and bacteriocins. However, acid production of LAB is the most important mechanism that inhibit bacterial pathogens. Weak acids can also get through the plasma membrane of a bacterial cell and breakdown into ions in a high pH environment, causing cytoplasmic acidification. In an acidic environment, enzymes are damaged, protein synthesis is inhibited, genetic material is destroyed, nutritional absorption is disrupted, and the substructure and function of cell walls and membranes are damaged. Besides, organic acids exhibited multiple inhibitory activities such as energy competition, intracellular anion accumulation (which increases intracellular osmotic pressure), membrane effects, inhibition of biomacromolecule synthesis, induction of host cell to produce antimicrobial peptides, and intracellular pH effects.

Interestingly, pH in the control treatment of *L. innocua* CIP 80.11^T^ (LI) (Fig. 3B) decreased considerably at 6 h of incubation and remained relatively steady around 5 until the completion of the experiments, with no effect on its growth. This could explain that the genus *Listeria* can produce acid from glucose [58, 59]. *L. innocua* CIP 80.11^T^ was used as a nonpathogenic surrogate for *L. monocytogenes*. It has been reported that acid adaptation of *L. monocytogenes* is believed to be crucial for its survival rate, which results in the persistence of this pathogen in food-processing environments. *L. monocytogenes* growing in a mild acid environment could increase its resistance to the lethal pH condition in the stomach, and this phenomenon has been called acid tolerance response (ATR). With a pH range of about 4.5-6.0 at 30°C or 37°C, lactic and acetic acids can efficiently trigger the ATR of *L. monocytogenes* [60]. This was in line with Smith *et al*. [61], who stated that *L. monocytogenes* has a number of processes that enable it to cope with acidic conditions, such as those found in acidic foods and the gastrointestinal tract. As a result, while a mildly acidic environment did not suppress the growth of *L. innocua* CIP 80.11^T^, other chemicals such as H_2_O_2_, CO_2_, diacetyl, acetaldehyde, D-isomers of amino acids, reuterin, and bacteriocins may have an effect [62-65].

### Survival of Selected LAB under Simulated Gastrointestinal Tract (GIT) Condition

Selected LAB (C707, G502, and V202) were evaluated for survival in simulated stomach conditions by exposing washed cell suspensions to simulated gastric juice (pH 3) containing pepsin at 39°C for 3 h, mimicking the condition of the stomach. In this condition, they had low survival rates (Table 4). After 3 h in simulated stomach condition, their survival rate in simulated intestinal juice (pH 8) was then tested for 3 h at 39°C in the presence of ox bile salts, pancreatin, trypsin, and α-chymotrypsin. Only G502 was determined to be able to survive, whereas the others did not (Table 4).

One of the conditions for considering probiotic bacteria is the survival of LAB strains at pH 3.0 after exposure for 2-3 h. In general, mortality in an acidic environment occurs when particular proteins and DNA are damaged due to the LAB's inability to maintain intracellular pH, and internal acidification has lowered the function of H^+^-ATPase, an enzyme responsible for regulating internal and external H^+^ concentrations. The tolerance of probiotic LAB to acidic conditions and bile salts containing enzymes (pancreatin, trypsin, and α-chymotrypsin) in the stomach and intestines is critical for survival in the gut and eliciting positive effects in the host [66, 67]. The hydrochloric acid (HCl) in gastric juice causes the pH of the animal or human stomach to range between 2.5 and 3.5. Importantly, probiotic LAB must survive in an acidic stomach environment in order to reach the small intestine and colonize the host [68]. Probiotics must be able to overcome the deleterious effects of bile salts in the small intestine in order to survive in gastrointestinal transit and transiently colonize the gut, according to Ruiz *et al*. [69]. Bile salts have significant antibacterial properties and can disrupt the structure and composition of the cell membrane and cell wall, as well as cause DNA damage. Probiotics, on the other hand, are mostly taken in the presence of food or feed. It has been widely reported that the use of food or feed components protects probiotic LAB, hence increasing probiotic viability in the GIT environment [70, 71]. Furthermore, because pre- or pro-microencapsulation of bacteria is commonly used to improve the survival rate of probiotic microorganisms during gastric transit as well as food or feed processing [72], the survival of these pre- or pro-microencapsulated selected LAB in simulated GIT conditions should be investigated further.

In conclusion, there were several good commercial probiotics available around the world, and the isolation of LAB was well publicized. Furthermore, several researchers are still looking for more effective probiotics. This study, however, is the first to reveal the presence of LAB in the cloaca of male indigenous gamecocks, vagina of goats, and anus of healthy baby goats originating from Phitsanulok, Thailand. The findings of our investigation suggest that selected LAB could be used as a biopreservative to minimize foodborne pathogenic bacteria. They could not, however, survive under GIT conditions. Encapsulation of these selected LAB has to be explored further in order to improve their survival rate under GIT conditions and food or feed production.

## Figures and Tables

**Table 1:** Growth media and growth conditions of foodborne pathogenic bacteria and related LAB used as indicators.

Indicator strains	Growth medium	Growth conditions (°C/h)	Anaerobic condition
*Escherichia coli* CIP 76.24	BHI	37°C/24 h	No
*Lactobacillus sakei* subsp. *sakei* JCM 1157	MRS	37°C/24 h	No
*Listeria innocua* CIP 80.11^T^	BHI	30°C/24 h	No
*Salmonella* Typhimurium CIP 58.58	BHI	37°C/24 h	No
*Staphylococcus aureus* CIP 76.25	BHI	37°C/24 h	No

**Table 2:** Antimicrobial activity of isolated LAB against foodborne pathogenic bacteria and related LAB.

Isolated LAB	Inhibition Zone (mm)				

	*E. coli* CIP 76.24	*L. sakei* subsp. *sakei* JCM 1157	*L. innocua* CIP 80.11^T^	*S*. Typhimurium CIP 58.58	*S. aureus* CIP 76.25
LAB isolated from cloaca of indigenous gamecocks					
C707	3.33 ± 0.58^a^	0.00 ± 0.00	6.67 ± 1.15	5.00 ± 0.00	0.00 ± 0.00
C804	3.67 ± 1.15	1.00 ± 0.00	6.00 ± 0.00	2.33 ± 0.58	0.00 ± 0.00
C901	3.00 ± 1.00	0.00 ± 0.00	5.33 ± 0.58	4.67 ± 0.58	0.00 ± 0.00
C902	3.33 ± 0.58	0.00 ± 0.00	6.00 ± 1.00	4.00 ± 1.00	0.00 ± 0.00
C915	3.00 ± 0.00	0.00 ± 0.00	6.00 ± 1.00	2.67 ± 0.58	0.00 ± 0.00
LAB isolated from anus of healthy baby goats					
G502	4.67 ± 0.58	1.00 ± 0.00	4.00 ± 3.46	9.33 ± 1.15	3.00 ± 1.00
G606	3.67 ± 0.58	1.67 ± 0.58	4.00 ± 3.46	4.33 ± 0.58	5.00 ± 1.73
G608	3.67 ± 0.58	1.00 ± 0.00	7.00 ± 1.00	4.00 ± 1.00	4.67 ± 1.53
G611	4.67 ± 0.58	1.00 ± 0.00	5.67 ± 0.58	6.33 ± 0.58	3.67 ± 2.08
LAB isolated from vagina of goats					
V202	3.33 ± 0.58	7.33 ± 0.58	8.33 ± 1.53	7.00 ± 1.00	4.00 ± 1.00
V204	2.67 ± 0.58	7.33 ± 0.58	6.67 ± 0.58	6.00 ± 1.00	2.67 ± 1.15
V206	2.33 ± 0.58	6.67 ± 0.58	3.33 ± 4.04	8.33 ± 1.53	3.67 ± 1.53
V502	4.00 ± 1.00	6.00 ± 1.73	9.00 ± 1.73	3.33 ± 5.77	2.33 ± 0.58
LAB used as positive control*					
CF1GI 14	8.00 ± 2.00	0.00 ± 0.00	10.67 ± 2.31	12.67 ± 2.31	3.33 ± 1.15
CM6CR 07	9.33 ± 3.06	3.33 ± 1.15	8.67 ± 2.31	14.67 ± 1.15	2.00 ± 0.00
CM6CR 11	10.67 ± 3.06	2.67 ± 1.15	8.67 ± 1.15	16.00 ± 0.00	2.00 ± 0.00

^a^Mean ± SD from triplicate determinations.

*LAB isolated from chicken gastrointestinal tract originating from Phitsanulok, Thailand [10].

**Table 3:** Molecular identity and morphological properties of selected LAB isolated from gamecock and goat originating from Phitsanulok, Thailand.

Selected LAB	Source	Catalase test	Gram stain	Shape	Strain	% Identity	GenBank accession number of ref. strains	GenBank accession number
C707	CIG	-	+	Cocci	*Enterococcus faecium*	98.28	LC005845.1	MW857160
G502	AHG	-	+	Cocci	*Enterococcus faecium*	99.77	MT052594.1	MW857159
V202	VG	-	+	Cocci	*Pediococcus acidilactici*	99.57	MH375410.1	MW857158

CIG = Cloaca of indigenous gamecock, AHG = Anus of healthy baby goat, VG = Vagina of goat

**Table 4:** Viable count of the selected LAB when incubated at 39°C in the presence of pepsin (3 mg/ml) at pH 3 for 3 h and pancreatin (1 mg/ml), trypsin (1 mg/ml), α-chymotrypsin (1 mg/ml), and ox bile salts (5 mg/ml) at pH 8 for 3 h.

Selected LAB	0 h (log CFU/ml)	3 h (stomach condition) (log CFU/ml)	3 h (intestinal juice) (log CFU/ml)
C707	9.47 ± 0.15^A^	2.52 ± 0.15^B^	0 ± 0.00^C^
G502	7.57 ± 0.13^A^	3.79 ± 0.09^C^	5.85 ± 0.56^B^
V202	8.79 ± 0.09^A^	4.80 ± 0.11^B^	0 ± 0.00^C^

*Mean ± SD from triplicate determinations.

Different superscripts in the same row indicate significant differences (*p* < 0.05).
